# Occupational Noise-Induced Hearing Loss in Saudi Arabia: A Systematic Review

**DOI:** 10.7759/cureus.103463

**Published:** 2026-02-12

**Authors:** Fawziah A Roublah, Reem F Alnemari, Zeyad H Althumali, Elham M Qanadeely, Duja N Zarnogi, Abdullah H Jabbad

**Affiliations:** 1 Preventive Medicine, King Abdulaziz Medical City, Ministry of National Guard Health Affairs, Jeddah, SAU

**Keywords:** hearing loss, noise-induced hearing loss, occupational diseases, occupational noise-induced hearing loss, saudi arabia

## Abstract

Occupational noise-induced hearing loss (ONIHL) remains one of the most frequently reported occupational diseases worldwide. It occurs with epidemiologically significant frequency among workers exposed to hazardous noise levels and is largely predictable and preventable through effective workplace control measures. This review aimed to examine the evidence related to ONIHL in Saudi Arabia. A comprehensive search was conducted using major databases, including PubMed, Scopus, Google Scholar, Web of Science, and ScienceDirect, to identify relevant literature. A total of seven cross-sectional studies were included, involving 1,481 participants from different regions of Saudi Arabia. The prevalence of ONIHL varied significantly across occupational groups, ranging from 2% to 71.6%, with the highest rates reported among military personnel. This considerable variation is primarily attributable to differences in diagnostic methods. Across all studies, awareness of ONIHL and the use of hearing protection were generally poor. Many participants demonstrated limited knowledge and negative attitudes toward hearing conservation, particularly in high-risk environments such as dental settings and military operations. This review highlights a concerning combination of high prevalence rates of ONIHL and poor knowledge, attitudes, and preventive practices among the Saudi population. Age, years of professional experience, and occupational noise exposure were all substantially correlated with ONIHL, suggesting an association between prolonged exposure and hearing impairment. These demographic and occupational risk factors underscore the urgent need for proactive and targeted prevention strategies.

## Introduction and background

Globally, hearing loss resulting from occupational noise exposure is a serious health issue [1]. Noise-induced hearing loss is a prevalent occupational illness. Estimates suggest that noise exposure causes hearing loss in 1.3 billion people, with approximately 16% of adult cases of disabling hearing loss worldwide attributed to workplace noise exposure [2]. According to a review by Chen et al., occupational noise-induced hearing loss (ONIHL) is one of the most frequently reported occupational diseases, particularly in less developed regions, and its burden varies widely across regions and occupations globally, ranging from 11.2% to 58% [3]. Men are more adversely affected by occupational noise than women [1].

Hearing loss caused by noise can be either transient or permanent. Transient hearing loss occurs after short-term exposure to loud noise and typically returns to normal after a period of rest. In most cases, long-term exposure to loud noise gradually results in irreversible damage [4]. Depending on the type and intensity of exposure, noise can damage the ear through two primary mechanisms. The first mechanism involves exposure to extremely loud sounds exceeding 140 dB for a short duration. This overstretches the sensitive inner ear tissues beyond their elastic limits, causing tears. This form of injury, known as acoustic trauma, leads to immediate and irreversible hearing loss. The second mechanism involves prolonged or repeated exposure to noise levels ranging from 90 to 140 dB, which damages the cochlea metabolically rather than mechanically. The extent of this damage depends on the intensity and duration of exposure [5]. Prior research has demonstrated that individuals working in construction, manufacturing, mining, agriculture, utilities, and transportation, as well as those in the armed forces and music industry, are more susceptible to ONIHL [6].

Noise-induced hearing loss reduces quality of life by obstructing communication and increasing social isolation. In addition to limiting social interaction, it can cause social stress, depression, low self-esteem, impaired self-identity, and poor interpersonal relationships [6]. The risk of dementia is twice as high in older adults with mild hearing loss and five times higher in those with severe hearing loss [7]. Earlier research has also demonstrated a link between increased noise exposure and higher morbidity and mortality from cardiovascular disease [8]. Estimates indicate a 10-20% higher mortality rate among individuals with hearing loss [9]. Noise exposure may activate the endocrine and autonomic nervous systems, potentially increasing the risk of heart disease, stroke, and hypertension [10]. Furthermore, the effects of occupational noise exposure impose significant financial and health burdens on both individuals and society. In the United States, ONIHL is estimated to result in compensation payouts totaling $242.4 million annually [2]. These costs underscore the urgent need to address ONIHL and implement effective prevention strategies.

Noise-induced sensorineural hearing loss is permanent and irreversible, resulting from damage to cochlear hair cells, which lack regenerative capacity in humans. While there is no effective treatment to reverse hearing loss, noise-induced hearing loss is fully preventable. The use of personal protective equipment, such as earmuffs and earplugs, adherence to administrative controls (including restrictions on time spent in noisy environments), and implementation of engineering controls are all important strategies to reduce exposure to excessive noise [11].

Public health initiatives and workplace education are also essential to raise awareness and encourage protective practices. Regular audiometric testing helps detect early signs of hearing loss, allowing interventions before further damage occurs. Early identification is especially important for educational, occupational, and medico-legal purposes, particularly in high-risk professions such as construction, aviation, dentistry, and the military [10].

ONIHL remains a significant global public health concern, adversely affecting employees’ health, communication abilities, and productivity. Unfortunately, there is limited information available about ONIHL in Saudi Arabia. Given the ongoing transformation of the healthcare system in Saudi Arabia, it is imperative to understand the prevalence of ONIHL and its associated risk factors in order to mitigate its health and economic consequences. Therefore, this paper reviews the evidence related to ONIHL in Saudi Arabia, highlighting the crucial role of research in understanding and addressing this issue.

## Review

Methodology

This systematic review was conducted following the Preferred Reporting Items for Systematic reviews and Meta-Analyses (PRISMA) guidelines [12]. It has been registered in the international Prospective Register of Systematic Reviews (PROSPERO) with registration number CRD420251053837.

A comprehensive search was conducted using major databases, including PubMed, Scopus, Google Scholar, Web of Science, and ScienceDirect, to identify relevant literature, with only the first 200 results screened for Google Scholar. Additional studies were identified through a manual review of the reference lists of included articles. The search strategy incorporated Medical Subject Headings (MeSH) terms and free-text keywords, adapted for each database. Key search terms included “Occupational noise-induced hearing loss”, “Noise-induced hearing loss”, “Occupational diseases”, “hearing loss”, and “Saudi Arabia”.

The eligibility criteria were designed to include studies focusing on ONIHL in Saudi Arabia. To be included, studies had to be conducted between 2000 and 2024, published in English, freely accessible, and available in full text. Studies lacking a specific focus on ONIHL, as well as review articles, governmental reports, conference papers, animal studies, unpublished research, and opinion pieces, were excluded.

The study selection process followed a multistep approach to ensure the inclusion of relevant studies. In the first and second steps, titles and abstracts were screened for relevance by all research team members; duplicates and irrelevant articles were excluded. In the third step, full-text articles of potentially eligible studies were retrieved and assessed for inclusion. Microsoft Excel (Microsoft Corporation, Redmond, WA, USA) and EndNote (Clarivate, Philadelphia, PA, USA) were used to compile all search results, facilitating the identification of duplicates across databases.

Reviewers extracted key information from each included study, including the study title, design, year of publication, geographical location, number of participants, gender distribution, type of occupation, method of hearing loss diagnosis, prevalence of ONIHL, and other relevant outcomes. Summary tables were created to present key data from the selected studies, enabling comparison and qualitative evaluation of findings. The synthesis focused on identifying patterns, trends, and gaps in the literature to support the overall objectives of this systematic review, qualitatively synthesizing findings on hearing loss and its occupational risk factors without performing a quantitative meta-analysis.

The risk of bias was assessed using a modified version of the Newcastle-Ottawa Scale to evaluate the quality of the included cross-sectional studies across three domains: participant selection, comparability of the study, and outcome assessment. A scoring system with a maximum of 10 points was applied to reflect methodological rigor. Studies were classified into four levels: very good (9-10 points), good (7-8 points), satisfactory (5-6 points), and unsatisfactory (0-4 points) [13], and independently appraised by two reviewers. Discrepancies between reviewers were resolved through discussion or, when necessary, consultation with a third reviewer.

Results

Systematic Search Outcomes

**Figure 1:**
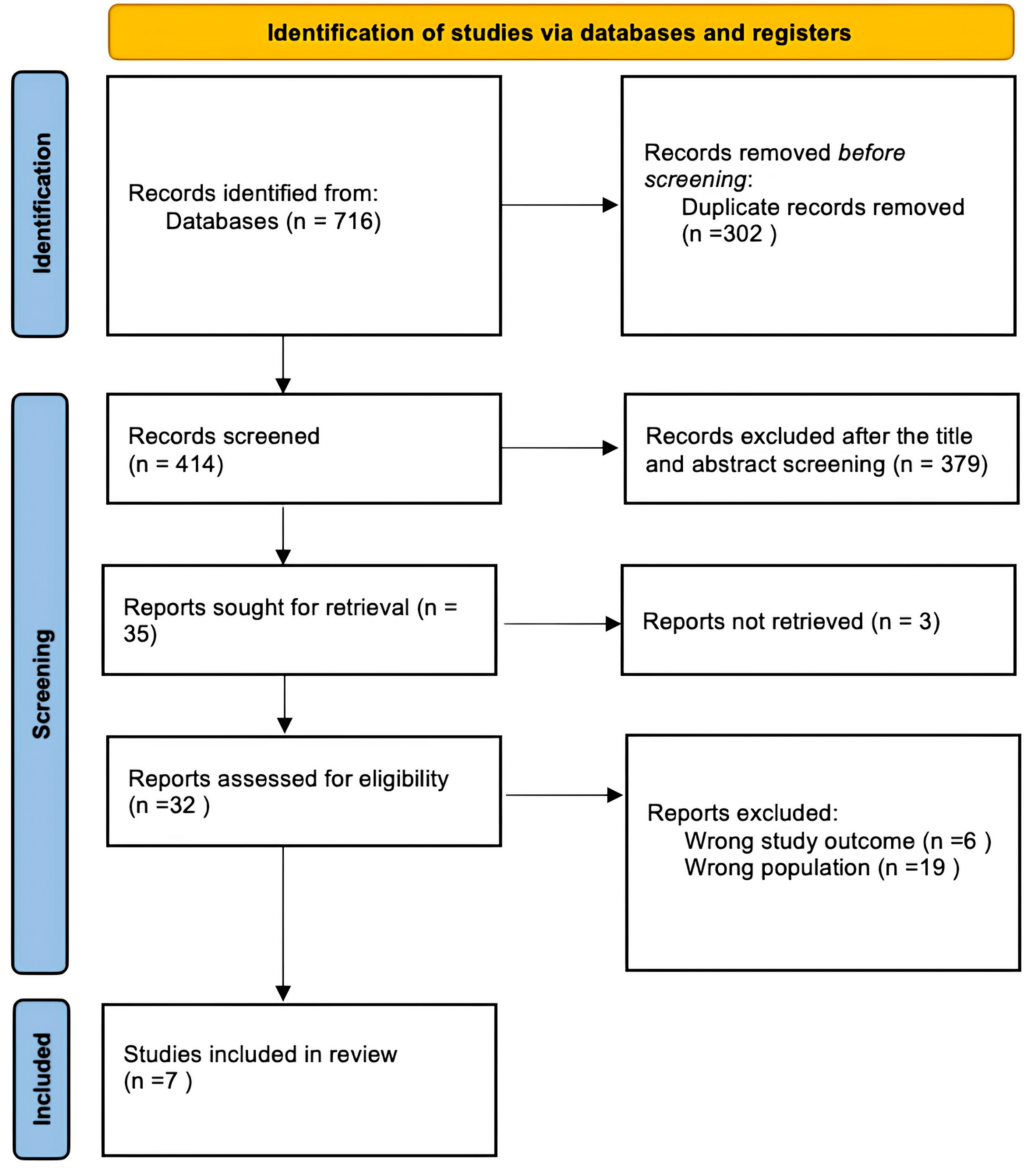
PRISMA diagram PRISMA, Preferred Reporting Items for Systematic reviews and Meta-Analyses

A total of 716 studies were identified through a systematic database search. After removing 302 duplicate records, 414 studies remained for title and abstract screening. Of these, 379 were excluded for not meeting the inclusion criteria, leaving 35 reports for full-text retrieval, of which three could not be accessed. The remaining 32 articles underwent full-text assessment for eligibility. Of these, 25 were excluded: six due to irrelevant outcomes and 19 for focusing on the wrong population. Ultimately, seven studies met the inclusion criteria and were included in this systematic review. The study selection process is summarized in Figure 1.

Sociodemographic Features of the Included Studies

All seven studies employed a cross-sectional design. The sociodemographic characteristics of the included studies are presented in Table 1. The studies were conducted across various regions of Saudi Arabia [14-20]. Specifically, two studies were conducted in Riyadh [18,20], one in Abha [14], one in Dhahran [19], one in Al Ahsa [17], and two studies did not specify a location [15,16]. The total number of participants across these studies was 1,481. Among 1,361 participants with reported gender, 1,041 (76.5%) were male, and 320 (23.5%) were female. Participant ages ranged from 18 to 60 years. The earliest study was published in 2016 [20], and the most recent studies were published in 2023 [15].

**Table 1:** Sociodemographic attributes of the participating populations NM, not mentioned

Study	Study design	City	Participants	Age range	Males (%)
Vaddamanu et al. (2023) [14]	Cross-sectional	Abha	120	20-55	NM
Alqarny (2023) [15]	Cross-sectional	Not specified	412	19-25	262 (63%)
Alhaider et al. (2023) [16]	Cross-sectional	Not specified	100	20-50	57 (57%)
Alsaab et al. (2021) [17]	Cross-sectional	Al Ahsa	409	20-60	409 (100%)
Alnemare (2019) [18]	Cross-sectional	Riyadh region	252	18-59	140 (55.6%)
Al-Omari et al. (2018) [19]	Cross-sectional	Dhahran	150	23-55	150 (100%)
Alabdulwahhab et al. (2016) [20]	Cross-sectional	Riyadh	38	25-40	23 (60.5%)

Prevalence of ONIHL in Saudi Arabia

The prevalence of ONIHL, diagnostic methods, and associated awareness, attitudes, and protective practices reported in the included studies are summarized in Table 2.

**Table 2:** Clinical features and results of the included studies NIHL, noise-induced hearing loss; NM, not mentioned; OAE, otoacoustic emission; ONIHL, occupational noise-induced hearing loss; PTA, pure-tone audiometry

Study	Population type	Hearing loss diagnosis	Hearing loss prevalence (%)	Awareness about ONIHL	Main outcomes
Vaddamanu et al. (2023) [14]	Dental technicians	Otoscopic evaluation, Weber test, OAE, and PTA	NM	NM	As working experience increased, a statistically significant threshold shift from 4,000 to 6,000 Hz was observed, suggesting that the noisy dental environment contributes to sensorineural hearing impairment.
Alqarny (2023) [15]	Medical students	NM	NM	The level of awareness about NIHL among medical students was 59.32%.	Access to relevant information and personal or family experience with hearing problems were strong predictors of awareness, indicating the need for improved education and preventive strategies regarding occupational noise risks.
Alhaider et al. (2023) [16]	Dental students, interns, and practitioners	Online application: mini hearing test	2 (2%)	The majority of dental professionals did not use hearing protective devices and did not consider dental noise exposure as a cause of tinnitus or hearing loss, indicating limited awareness of ONIHL.	A statistically significant relationship (p < 0.05) was found between dental specialty and the level of hearing loss.
Alsaab et al. (2021) [17]	Military personnel	PTA	71.60%	None of the participants used appropriate hearing protection, and more than half (54.3%) were unaware of the risks of prolonged noise exposure. Only 23% had previously undergone hearing evaluation, and 5.9% completed annual hearing examinations.	ONIHL is a common occupational disability among military personnel, highlighting the need for hearing conservation programs to ensure hearing protection use and manage noise exposure effects.
Alnemare (2019) [18]	Dental students and practitioners	NM	NM	Knowledge and practice scores were weak, while attitude scores were average.	Knowledge, attitude, and practice scores were significantly influenced by age, educational level, center designation, nationality, daily occupational noise exposure, and years of study and practice.
Al-Omari et al. (2018) [19]	Military pilots	PTA	28 (18.4%)	NM	Pilots operating fixed- and rotary-wing aircraft were highly susceptible to hearing impairment, with fixed-wing pilots and those with more than 2,000 flight hours at greater risk of NIHL.
Alabdulwahhab et al. (2016) [20]	Dental practitioners	PTA	15.80%	NM	Dental clinics were associated with hearing impairment, with the left ear more affected than the right. Dentists experienced NIHL more frequently than the control group.

The reviewed literature includes studies from various occupational groups in Saudi Arabia exposed to noise, including dental technicians [14], dental practitioners [16,18,20], medical students [15], dental students [16,18], and dental interns [16].

The military field was also studied in Saudi Arabia. Al-Omari et al. focused on military pilots [19], while Alsaab et al. included other military personnel [17]. The studies revealed a wide range in the prevalence of hearing loss, from 2% to 71.6%, depending on the type of occupation and the diagnostic method used [16,17]. Military personnel, including pilots, were among the most affected groups, with prevalence rates ranging from 18.4% to 71.6%. In both studies, hearing loss was diagnosed using pure-tone audiometry (PTA) [17,19]. Notably, fixed-wing pilots with over 2,000 flight hours were found to be at higher risk of noise-induced hearing loss [19].

Among dental professionals, prevalence ranged from 2% in a sample assessed using an online hearing evaluation [18] to 15.8% when PTA was employed as a diagnostic method [20]. One study investigating dental laboratory employees reported mild hearing impairment via PTA, with the left ear more severely affected. Additionally, otoacoustic emission testing indicated that 15.5% of participants failed screening in the left ear and 17.3% in the right ear, with those participants referred to an ear specialist [14]. The remaining studies [15,18] were not designed to measure prevalence.

Awareness, Attitudes, and Protective Practices Toward ONIHL

In Saudi Arabia, ONIHL is not only prevalent but also associated with gaps in awareness, attitudes, and protective behaviors. Alqarny assessed medical students’ knowledge of noise-induced hearing loss and found that 59.32% of participants demonstrated adequate awareness. Access to relevant information and personal or family experience with hearing problems were strong predictors of awareness [15]. Alnemare similarly assessed knowledge among dental practitioners and reported weak scores in knowledge and practice, with an average attitude score. Knowledge, attitude, and practice scores were significantly influenced by age, educational level, years of study and practice, country, and daily occupational noise exposure [18].

Regarding protective practices, Alhaider et al. found that dental professionals neither used hearing protection nor believed that noise in dental settings could cause auditory issues [16]. Attitudes toward ONIHL were generally negative: none of the participants used appropriate hearing protection, more than half were unaware of the risks of loud noise exposure, and few had previously undergone a hearing evaluation [17].

Quality Assessment 

All seven studies were independently appraised by two reviewers. Three studies scored between 5 and 6, and the remaining four scored between 7 and 8 out of 10, indicating satisfactory and good quality, respectively. Any discrepancies were resolved through consultation with a third reviewer (Table 3). Among the included studies, higher-quality studies (scores 7-8) generally reported ONIHL prevalence ranging from 15.8% to 71.6%, while lower-quality studies (scores 5-6) either reported very low prevalence (2%) or did not report prevalence. These findings suggest that methodological rigor may influence the reporting of ONIHL prevalence.

**Table 3:** Quality assessment of included studies using the Newcastle-Ottawa Scale

S. no.	Author (year)	Title	Study design	Selection (max five stars)	Comparability (max two stars)	Outcome (max three stars)	Total score (max 10 stars)	Quality of the study
1	Vaddamanu et al. (2023) [14]	Assessment of hearing performance of dental technicians due to the professional noise exposure	Cross-sectional	★★★★	★	★★	7	Good
2	Alqarny (2023) [15]	Noise-induced hearing loss and use of hearing protection awareness among medical students in Saudi Arabia: mixed qualitative and quantitative study	Cross-sectional	★★★	★	★	5	Satisfactory
3	Alhaider et al. (2023) [16]	Impact of noise on the hearing and tinnitus among dental students, interns, and dental practitioners: a cross-sectional study	Cross-sectional	★★★	★	★	5	Satisfactory
4	Alsaab et al. (2021) [17]	Hearing impairment in military personnel in Eastern Saudi Arabia	Cross-sectional	★★★★	★★	★★	8	Good
5	Alnemare (2019) [18]	Knowledge, attitude, and practice of dental students and practitioners toward noise-induced hearing loss in Saudi Arabia: a cross-sectional analytical study	Cross-sectional	★★★★	★	★	6	Satisfactory
6	Al-Omari et al. (2018) [19]	Association of flying time with hearing loss in military pilots	Cross-sectional	★★★★★	★	★★	8	Good
7	Alabdulwahhab et al. (2016) [20]	Hearing loss and its association with occupational noise exposure among Saudi dentists: a cross-sectional study	Cross-sectional	★★★★★	★	★★	8	Good

Discussion

This systematic review identified and synthesized seven cross-sectional studies conducted in various locations across Saudi Arabia, investigating the prevalence, knowledge, attitudes, and practices related to ONIHL. The studies included multiple occupational groups, such as dental professionals, medical students, and military personnel.

The prevalence of ONIHL in Saudi Arabia ranged from 2% [16] to 71.6% [17], primarily depending on the occupation and the assessment methods used. For comparison, Zhou et al. reported a prevalence of 21.3% in China [21]. In the United States, workers in construction, manufacturing, mining, agriculture, utilities, transportation, military, and other sectors are at risk for ONIHL, with an estimated 20% of noise-exposed workers experiencing permanent hearing impairment [22]. Similarly, manufacturing, transportation, mining, and agricultural sectors are major sources of noise exposure in Asia [23,24].

Military personnel, including pilots, exhibited the highest prevalence rates of ONIHL [17,19]. This finding is expected, as military personnel are frequently exposed to dangerously high noise levels. In the United States military, hearing loss is the most common occupational health impairment among service members [25]. Moreover, tinnitus and hearing loss rank as the first and second most common service-connected disabilities, respectively, with estimated annual compensation of $242.4 million [26]. Age was also identified as a statistically significant risk factor for ONIHL in both cited studies of military personnel [17,19].

Conversely, lower prevalence rates were observed among dental professionals, particularly in studies using online or self-reported assessments [16]. These lower rates may reflect underreporting or underdiagnosis due to less reliable measurement techniques. In contrast, studies employing PTA were more likely to detect subclinical hearing abnormalities [20], emphasizing the need for standardized diagnostic tools in future research.

Despite the lower reported prevalence, dental procedures generate considerable noise, including deflasking casting rings, steam and compressed air cleaning of workpieces, and the fabrication of crowns, bridges, and removable partial dentures [27]. High sound pressure levels are also produced by sandblasters, dental vibrators, and polishing machines, reaching approximately 86 dB when processing removable partial dentures, 90 dB with steam jets, and 105 dB with compressed air blasting. These measurements are based on either equipment runtime or an eight-hour exposure period [27-29].

Assessment of knowledge, attitudes, and practices regarding ONIHL in Saudi Arabia revealed significant gaps, with evidence primarily from military and dental personnel, while most other occupational sectors remain unrepresented. Among medical students, 59.32% demonstrated adequate knowledge, with awareness influenced by personal experience or access to educational resources [15]. Dental practitioners and students showed weaker knowledge and poor preventive practices, despite exposure to high-frequency dental equipment known to cause progressive hearing loss [18]. Medical students and other healthcare workers play a key role in promoting hearing protection and providing accurate guidance on hearing health. Limited access to information, societal norms that do not prioritize hearing protection, and inadequate education are key contributors to this lack of awareness.

Attitudes toward hearing protection were particularly negative in military settings, where personnel are exposed to high-decibel noise from machinery, weapons, and aircraft. According to Alsaab et al., over half (54.3%) of military personnel were unaware of the risks associated with prolonged noise exposure, and none used hearing protection. Compliance with annual screening was very low, and routine hearing examinations were rarely conducted [17]. Poor adherence to hearing tests likely contributes to the high prevalence observed. These findings highlight widespread neglect of preventive measures, largely due to limited awareness and insufficient training.

Knowledge, attitude, and practice scores were influenced by demographic and occupational factors such as age, years of experience, education level, and daily duration of noise exposure, suggesting that targeted interventions could be more effective if tailored to specific groups [18].

These results underscore the importance of implementing occupational hearing conservation programs, particularly in high-risk industries such as dentistry and the military. Routine audiometric screening, mandatory use of hearing protection, and targeted awareness campaigns should be prioritized. Incorporating ONIHL education into medical and dental curricula could promote early preventive behaviors. Institutional policies must ensure ongoing training, monitoring, and adherence to occupational safety standards regarding acceptable noise levels. Even among knowledgeable individuals, the lack of protective behaviors highlights a behavioral gap that requires attention through corporate culture changes and effective policy enforcement.

The systematic review included a limited number of studies, all cross-sectional in design. Despite this limitation, including these studies was necessary to provide a comprehensive overview of ONIHL in the available literature. Most studies used objective hearing assessments, such as PTA or otoacoustic emissions, while one study relied on self-reported data. The use of objective measures enhances the validity of both exposure and outcome data. Heterogeneity of outcome measures complicated cross-study comparisons. Publication bias is a possibility, as studies with favorable outcomes are often more easily published. Although job loss or relocation due to hearing impairment appears uncommon in most healthcare and technical professions, some selection bias may still exist. Overall, the limitations are minor and support the general reliability of the cross-sectional findings.

## Conclusions

This study highlights a concerning combination of high ONIHL prevalence and poor knowledge, attitudes, and preventive practices among the Saudi population. Age, years of professional experience, and occupational noise exposure were all strongly associated with ONIHL, suggesting a link between prolonged exposure and hearing impairment. The identified demographic and occupational risk factors emphasize the urgent need for proactive and targeted prevention strategies.
